# Assessment of prognosis and responsiveness to immunotherapy in colorectal cancer patients based on the level of immune cell infiltration

**DOI:** 10.3389/fimmu.2025.1514238

**Published:** 2025-02-03

**Authors:** Kaili Liao, Minqi Zhu, Lei Guo, Zijun Gao, Jinting Cheng, Bing Sun, Yihui Qian, Bingying Lin, Jingyan Zhang, Tingyi Qian, Yixin Jiang, Yanmei Xu, Qionghui Zhong, Xiaozhong Wang

**Affiliations:** ^1^ Jiangxi Province Key Laboratory of Immunology and Inflammation, Jiangxi Provincial Clinical Research Center for Laboratory Medicine, Department of Clinical Laboratory, The Second Affiliated Hospital, Jiangxi Medical College, Nanchang University, Nanchang, Jiangxi, China; ^2^ School of Public Health, Jiangxi Medical College, Nanchang University, Nanchang, Jiangxi, China; ^3^ The 2^nd^ Clinical Medical College, Jiangxi Medical College, Nanchang University, Nanchang, Jiangxi, China; ^4^ Queen Mary College, Jiangxi Medical College, Nanchang University, Nanchang, Jiangxi, China; ^5^ The 1^st^ Clinical Medical College, Jiangxi Medical College, Nanchang University, Nanchang, Jiangxi, China

**Keywords:** colorectal cancer, immune-related gene, immune cell infiltration, WGCNA, prognostic model

## Abstract

**Objective:**

To build a new prognostic risk assessment model based on immune cell co-expression networks for predicting overall survival and evaluating the efficacy of immunotherapy for colon cancer patients.

**Methods:**

The Cancer Genome Atlas (TCGA) database was used to obtain mRNA expression profiling data, clinical information, and somatic mutation data from colorectal cancer patients. The degree of tumor immune cell infiltration of the samples was analyzed using the CIBERSORT algorithm. Co-expression of immune-related genes was analyzed using weighted correlation network analysis (WGCNA) and gene modules were identified. Prognosis-related genes were screened and models were constructed using LASSO-Cox analysis. The models were validated by survival analysis. The prognostic potential of the models was quantitatively assessed using Cox regression analysis and the development of column line plots. Immunotherapy sensitivity analysis was performed using CIBERSORT and TIMER algorithms. Gene biofunction analysis was performed using Gene set enrichment analysis (GSEA) and Gene set variation analysis (GSVA). And the chemotherapeutic response to different drugs was assessed.

**Results:**

We established a novel prognostic model utilizing the WGCNA method, which demonstrated robust predictive accuracy for patient survival. The high-risk subgroup in our model exhibited elevated immune cell infiltration coupled with a higher tumor mutation burden, but the difference in response to immunotherapy was not significant compared to the low-risk group. Furthermore, we identified distinct chemotherapy responses to 39 drugs between these risk subgroups.

**Conclusion:**

This study revealed a significant correlation between high levels of immune infiltration and unfavorable prognosis in patients with colon cancer. Furthermore, an accurate prognostic risk prediction model based on the co-expression of relevant genes by immune cells was developed, enabling precise prediction of survival of colon cancer patients. These findings offer valuable insights for accurate prognostication and comprehensive management of individuals diagnosed with colon cancer.

## Introduction

Colorectal cancer (CRC) is not only the third most prevalent cancer worldwide but also ranks second in cancer-related deaths (1). Despite significant advancements in medical science and technology that have improved the five-year survival rate, the prognosis for CRC remains suboptimal. Several factors, including patient age, lymph node metastasis, tumor size, degree of differentiation, and clinical staging significantly impact disease prognosis (2, 3). Traditional TNM staging provides predictive information for cancer prognosis but fails to anticipate treatment response due to its limited consideration of the host’s immune response (4, 5). Hence, identifying new biological markers for prognosis and treatment prediction is crucial for clinical decisions and enhancing CRC outcomes. In the view of existing studies, immune cells not only play an important role in the occurrence and development of colorectal cancer (6) but also are closely related to the prognosis of patients (7). Tumor cells adeptly manipulate immune cell activity to evade immunosurveillance, thereby facilitating their growth and dissemination (8). Consequently, investigating immune cells holds paramount significance for devising innovative immunotherapeutic strategies and appraising patient prognostic outcomes. CIBERSORT is a computational algorithm that is widely used for estimating the abundance of different cell types in a mixed cell population based on gene expression data. It enables accurate estimation of immune cell fractions in tumor tissues (9). CIBERSORT can accurately estimate the relative proportions of various immune cells (such as T cells, B cells, macrophages, dendritic cells) in colorectal cancer samples, thereby helping researchers understand the interaction between the immune system and tumor cells (10). And by analyzing the infiltration patterns of immune cells identified by CIBERSORT, researchers can develop potential prognostic biomarkers (11).

Infiltration of various subpopulations of immune cells in the tumor microenvironment has different effects on patient prognosis. According to Galon et al., there is a strong association between increased infiltration of CD8+ T cells, heightened expression of PD-L1, and improved survival rates. Conversely, lower levels of CD8+ T cells and PD-L1 expression are linked to poorer outcomes (12). Clinically, the use of immune checkpoint inhibitors in tumor treatment has been shown to significantly enhance therapeutic efficacy, despite variations in individual immune responses (13). The identification of optimal immune-related inhibitors remains a formidable challenge. Thorsson et al. underscore the utility of immune-related gene expression data across diverse cancer types for classifying and prognosticating cancer patients (13). These studies offer novel avenues for immunotherapeutic exploration. Despite affirming the critical role of immune-related gene expression levels in predicting colorectal cancer patient prognosis, a deficiency exists in comprehensive research applications assessing patient survival and immune therapy responses based on these immune cell-related genes. Therefore, it is necessary to conduct additional research in order to confirm and validate the role of co-expressed genes related to immune cells in evaluating the prognosis of colorectal cancer. This will help in developing more accurate personalized treatment approaches. WGCNA is a systematic approach utilized for analyzing complex biological data with multiple dimensions. This method not only simplifies the detection of possible associations in gene co-expression but also offers valuable perspectives for uncovering potential biological indicators. In the field of colorectal cancer investigation, WGCNA demonstrates significant advantages in predicting tumor invasiveness and patient survival. Lv et al. employ WGCNA and the least absolute shrinkage and selection operator (LASSO) algorithm to discover three genetic patterns linked to cancer-associated fibroblasts that may be potential CRC prognostic biomarkers (14). Cheng et al. identify three gene prognosis prediction models based on the result of WGCNA from the TCGA cohort which were developed to estimate the survival rates of bladder cancer patients (15). Indeed, these studies contribute significantly by providing valuable insights into investigating immune-related genes in CRC. These insights help detect the invasiveness of tumors and assess prognosis, thereby offering promising avenues for further research.

Our study employed the CIBERSORT algorithm to annotate the transcriptome sequencing results obtained from TCGA’s CRC samples. This enabled us to identify distinct subgroups of infiltrated immune cells within the tumor, thus enhancing our understanding of its immunological characteristics. The WGCNA algorithm was employed to cluster and select genes expressing differentially in these immune cells, resulting in the identification of a feature gene set associated with differential immune cell characteristics. COX analysis was then applied to filter prognostically relevant target genes from this feature gene set. Subsequently, a prognosis risk assessment model was established by combining the lasso algorithm with CRC patient prognosis information. Through testing with external datasets, our study verified that the model has good prognostic assessment efficacy. In addition, the study investigated the relationship between the risk score of the model, tumor mutation burden, and infiltration of immune cells. Enrichment analysis was performed to investigate potential biological functions. Finally, by comparing the IC50 of patients with different risks, this study deciphers the differences in sensitivity of patients with different immune characteristics to different drugs

## Materials and methods

### Study objects

We acquired mRNA expression profiling data, along with clinical information and somatic mutation data, from the TCGA database (https://portal.gdc.cancer.gov/) for individuals diagnosed with colorectal cancer. The dataset encompassed 473 patients with CRC and included 41 samples representing normal tissue. Sample data for the test set were generated by selecting a dataset containing at least 30 colorectal cancer samples from the GEO database, and the dataset ID GSE17536 was ultimately selected, containing a total of 177 samples. Analysis of somatic mutation data and visualization of the results were achieved with the Maftools R software package.

### Examination of the infiltration of immune cells

We employed the CIBERSORT bioinformatics algorithm for assessing the cellular makeup of complex tissues using standardized gene expression profiles. This allowed us to assess 22 immune cells that infiltrate tumors in our samples. We employed the Immune Plot R package to visualize this infiltration, while barplots were used to display the degree of immune cell infiltration in colon cancer patients. Corheatmap was utilized for processing and visualizing correlations between these immune infiltrating cells, and heatmaps were generated to demonstrate the infiltration levels of different immune cells between normal samples and colon cancer patients.

### Immune cell-associated co-expression network analysis

The analysis of co-expression patterns among immune-related genes in the samples was conducted using the WGCNA R package. The assessment of average connectivity was conducted using scale-free topological complementary models and various soft threshold powers. The WGCNA algorithm was utilized to identify gene modules that are co-expressed. Modules exhibiting stronger correlations were selected for further analysis by identifying genes with the most significant associations.

### Risk scoring model construction

We integrated RNA-seq data from colon cancer patients to analyze the expression of immune-related genes and their correlation with clinical outcomes. By conducting one-way Cox regression analysis, we identified immune-related genes that were associated with patient prognosis. We initially selected potential prognostic markers based on significant differences in gene expression (*P*<0.05). To further refine our selection process, we employed LASSO regression with penalty parameter estimation using 10-fold cross-validation and multifactorial Cox regression analysis. This approach allowed us to develop a precise risk model specifically focused on immune genes, enabling accurate prediction of patient outcomes. Finally, we calculated each patient’s risk score by considering the gene expression levels determined through multivariate Cox regression analysis, following the equation: risk score =∑(coef*gene expression), “coef” refers to the correlation coefficient of the corresponding gene.

### Validation analysis of the prediction model

After constructing the model, we utilized the TCGA and GEO databases to define the training set and validation set, respectively, for model validation. To assess the disparities in survival rates among these cohorts, we conducted a log-rank test for survival analysis and generated Kaplan-Meier curves accordingly. In order to further validate the model, we employed the riskPlot R package to generate risk heatmaps, patient risk score curves, and patient survival bubble plots. These visualization techniques allowed us to explore gene expression patterns in patients at different risks and to observe actual differences in patient survival. Independent prognostic analyses were achieved by univariate and multivariate Cox regression analyses, while the predictive accuracy of our constructed models was compared at different survival periods using joint ROC analyses. Additionally, we evaluated how our model’s accuracy compared with other clinical indicators. Furthermore, we assessed the correlation between prognosis-related genes involved in our constructed model by conducting Survival analyses using the geneSurvival R package followed by obtaining Kaplan-Meier curves through Log-rank test.

### Creation of a nomogram utilizing a risk score system

We developed a predictive nomogram for estimating the overall survival (OS) of patients at 1, 3, and 5 years using the Survival and RMS R software packages. The nomogram integrated various scoring factors including gender, age, stage, risk score, and other variables identified as independent risk factors through Cox regression analysis. By collectively assessing these scoring factors, we quantitatively evaluate the prognostic potential of our immune-related genetic risk scoring model for practical clinical application. In addition, we evaluated the dependability of the column plots through the calibration of calibration curves.

### Clinical relevance analysis

Differences in clinicopathologic characteristics between high-risk and low-risk groups were assessed using the chi-square test. The analysis was conducted utilizing pheatmap and ggpubr R software packages, with the results visualized through heat maps. Clinical characteristics considered included age (≤65 years vs >65 years), gender (male vs female), tumor grade (G1, G2, and unknown), T status (T1-T4), N status (N0-N2), and M status (M0, M1, and unknown). Furthermore, stratified analyses were performed to compare variations in tumor grading between the high-risk and low-risk groups.

### Somatic mutation analysis and tumor microenvironment analysis

In this study, the VarScan2 pipeline was utilized to compute the tumor mutation burden (TMB) for each specimen, for which the VarScan2 annotation file obtained from the TCGA database was used. The relationship between TMB and risk scores was evaluated using Spearman correlation analysis, which measures rank correlation. To visually represent genes with elevated mutation frequencies, we employed the maftools R software package to generate a waterfall plot showcasing the top 20 genes. To depict the disparities and correlations of TMB between the two risk groups, we utilized box plots and scatter plots created via R software. Kaplan-Meier plots were generated to display patient survival based on their levels of high or low TMB, as well as stratified analyses comparing patients’ TMB against their respective risk scores. In order to quantify stroma and immune cell content within the tumor microenvironment for each colon cancer patient, we applied the ‘ESTIMATE’ algorithm. Revealed in the violin plots were variations in the stromal, immune cell, and tumor purity scores between the high- and low-risk groups.

### Immunotherapy sensitivity analysis

The relationship between the genes involved in constructing models and immune cells was evaluated using CIBERSORT and TIMER algorithms, with the results presented as scatter plots. Multiple software algorithms, such as XCELL, TIMER, QUANTISEQ, MCPCOUNTER, EPIC, CIBERSORT-ABS, and CIBERSORT were utilized to predict associations between different immune cells and risk scores. These results were then visualized and analyzed through bubble plots. The potential association between the genes utilized in constructing the model and 45 immune checkpoint-related genes was visually represented using a heat map, aiming to investigate their correlation with the therapeutic efficacy of immune checkpoint blockade (ICB). To illustrate the variation in response to immunotherapy between groups at high risk and low risk, a violin plot was employed.

### Bifunctional analysis and chemotherapy response analysis

The enrichment analysis of genes in various pathways was conducted using GSEA to identify potential regulatory substrates. Furthermore, GSVA analysis was performed to validate the model by assessing the correlation between different functions or pathways and model genes. The pRRophetic R software package was utilized to determine the semi-inhibitory concentration (IC50) of various chemotherapy drugs. A total of 39 drugs were screened from the Genomics of Drug Sensitivity in Cancer (GDSC) dataset, and a comparison was made between the IC50 values of the two groups to assess differences in drug sensitivity. The findings are presented using boxplots, with statistical significance set at *P*<0.05 for detecting variations between the two groups.

## Result

### Immune cell infiltration analysis

**Figure 1:**
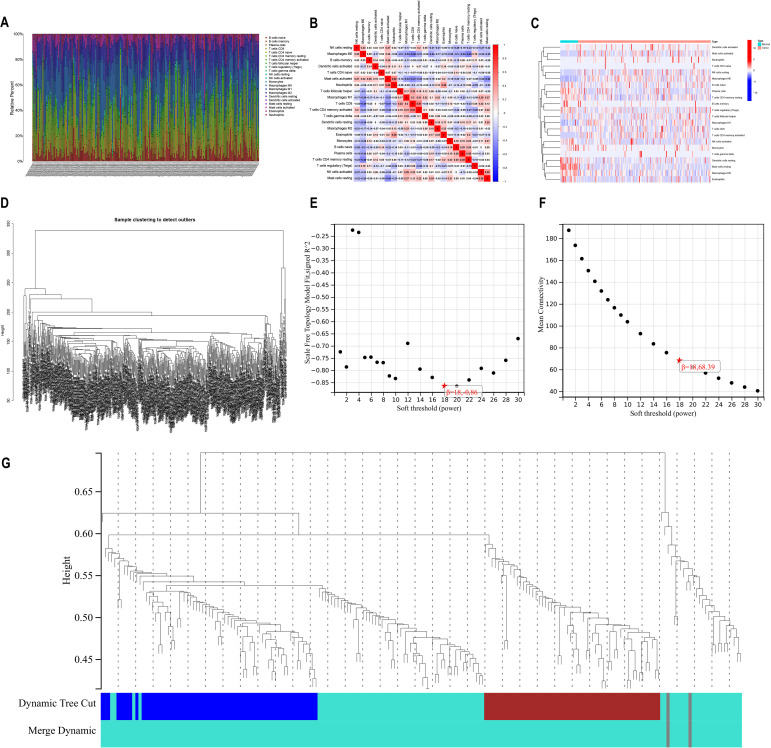
Immune infiltration analysis versus WGCNA analysis **(A)** A bar chart illustrating the distribution of 22 immune cell types obtained from analyzing immune cell infiltration in 514 samples of bowel cancer. The visualization showcases the varying levels of immune cell infiltration observed in each sample. **(B)** Heatmap displaying the correlation among 22 different types of immune cell infiltration. **(C)** Heatmap illustrating variations in the infiltration levels of 22 immune cell types. The horizontal axis represents increasing risk from left to right. **(D)** Dendrogram for gene clustering analysis **(E)** Scale-free topological complementary model analysis suitable for various soft-threshold powers **(F)** Analysis of average connectivity for different soft-threshold powers **(G)** Gene dendrogram and clustering module colors.

We collected data on immune cell infiltration analysis from the TCGA database, including 514 colon cancer patients (**Figure 1A**). Samples were visually depicted using stacked histograms to showcase the extent of infiltration by immune cells. The relationships among the 22 immune-cell infiltrations were visualized in a correlation heat map (**Figure 1B**). A visual representation of the infiltration patterns of 22 immune cells (**Figure 1C**) revealed increased levels of activated mast cells, neutrophils, resting natural killer cells, M0 macrophages, M1 macrophages, and activated CD4 memory T cells in tumor samples. Conversely, normal samples exhibited decreased levels of resting dendritic cells, resting mast cells, M2 macrophages, and eosinophils compared to the tumor samples.

### Construction of weighted gene co-expression networks to analyze co-expression model

The WGCNA method was employed to establish co-expression networks for clustering immune cell-related genes. **Figure 1D** demonstrates the sample division and unsupervised class clustering analysis. We constructed the network for different soft threshold powers and drew the connectivity distribution diagram. In order to make the fitting degree reach 0.8 or above (**Figure 1E**) while maintaining high network connectivity (**Figure 1F**), we finally selected 18 as the appropriate power value. By calculating the co-expression correlation network based on gene expression levels, distinct clusters represented by unique colors were assigned to these genes. We found that immune cell-related genes were mainly clustered in the grey and turquoise modules (**Figure 1G**), indicating that these two modules had the highest correlation, so we selected the grey and turquoise modules for further analysis.

### Construction of prognostic models

**Figure 2:**
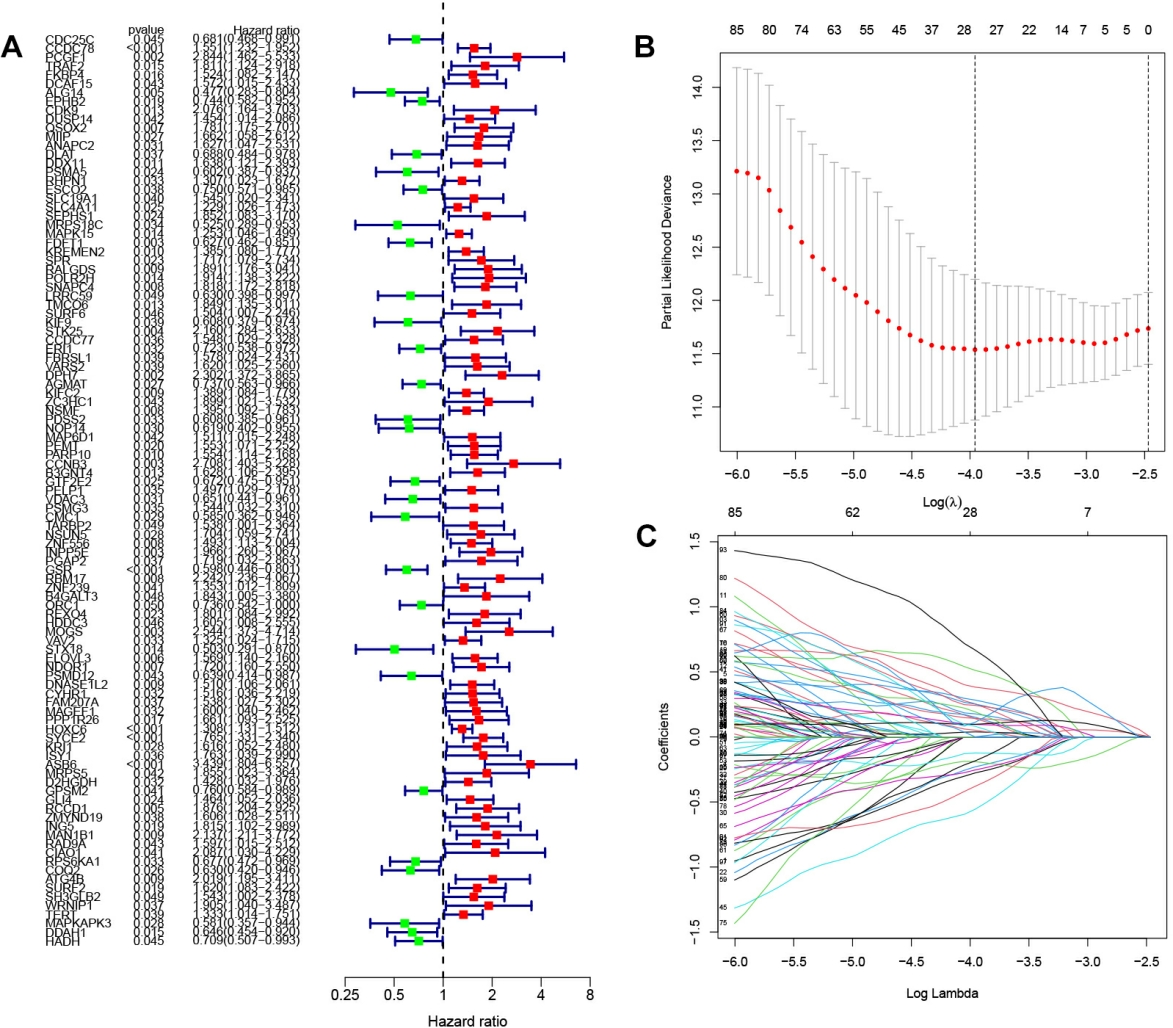
Screening of genes involved in constructing prognostic models **(A)** Forest plot illustrating the results of one-way analysis for 103 genes associated with prognosis. **(B)** K-fold cross-validation for parameter tuning in the LASSO model. The horizontal coordinate is Log(λ),and the vertical coordinate is the biased likelihood deviation **(C)** Spectrum of coefficients obtained through the LASSO method. Horizontal coordinates are Log Lambda values and vertical coordinates are gene coefficients.

After quantifying gene expression levels within their respective modules for each sample, we integrated this data with survival information. Furthermore, we merged the gene expression and survival data from TCGA and GEO databases to screen out the genes associated with prognosis. Initial identification of prognostically relevant genes was conducted through single-factor analysis (**Figure 2A**), yielding a total of 103 candidate genes. Subsequently, LASSO regression was performed to further screen the above candidate genes, and the lambda value with the smallest partial likelihood deviation was selected to determine the appropriate number of variables (**Figures 2B, C**). Twenty-eight genes were screened out by LASSO regression for Cox model construction. After multivariate Cox analysis, based on statistical significance (*P*<0.05), 14 immune cell-related genes significantly related to prognosis were finally selected to construct the risk scoring model. These selected genetic markers were utilized in developing our prognostic model which incorporates a specific formula for calculating individual patient risk scores: risk scores = (-0.426543178479338 * CDC25C expression) + (0.634483816982898* FKBP4 expression) + (0.550493338785946* DUSP14 expression) + (0.715265330715893* SLC19A1 expression) + (-0.588620522569632* MRPS18C expression) + (-1.03092755031287* NOP14 expression) + (0.567308117913096* CCNB3 expression) + (0.584860382870649* B3GNT4 expression) + (-0.471854560256281* ORC1 expression) + (-) 0.592801435620474*PSMD12 expression)+(0.587951048173452* SYCE2 expression)+(0.882739106800858*ISY1 expression)+(1.5180472208259* CIAO1 expression)+(-0.749492751586061* MAPKAPK3 expression)

### Validation of the prognostic model

**Figure 3:**
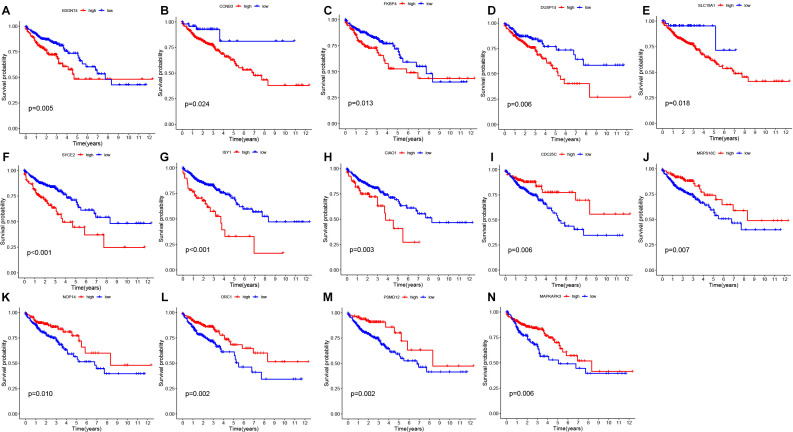
Survival analysis of prognostic genes **(A-N)** Survival plots derived from gene survival analyses of the 14 genes involved in model construction. The observation time is represented by the horizontal coordinate, while the survival rate is depicted on the vertical axis.

By survival analysis, we found that all 14 genes used in the model construction had a significant correlation with the prognosis of patients (*P*< 0.05). By analyzing the survival curves of these genes, we observed that patients with lower expression levels of B3GNT4, CCNB3, FKBP4, DUSP14, SLC19A1, SYCE2, ISY1, CIAO1 and other genes had longer OS than those with higher expression levels. On the contrary, patients with high expression of CDC25C, MRPS18C, NOP14, ORC1, PSMD12 and MAPKAPK3 had a better prognosis than those with low expression. (**Figure 3**)

**Figure 4:**
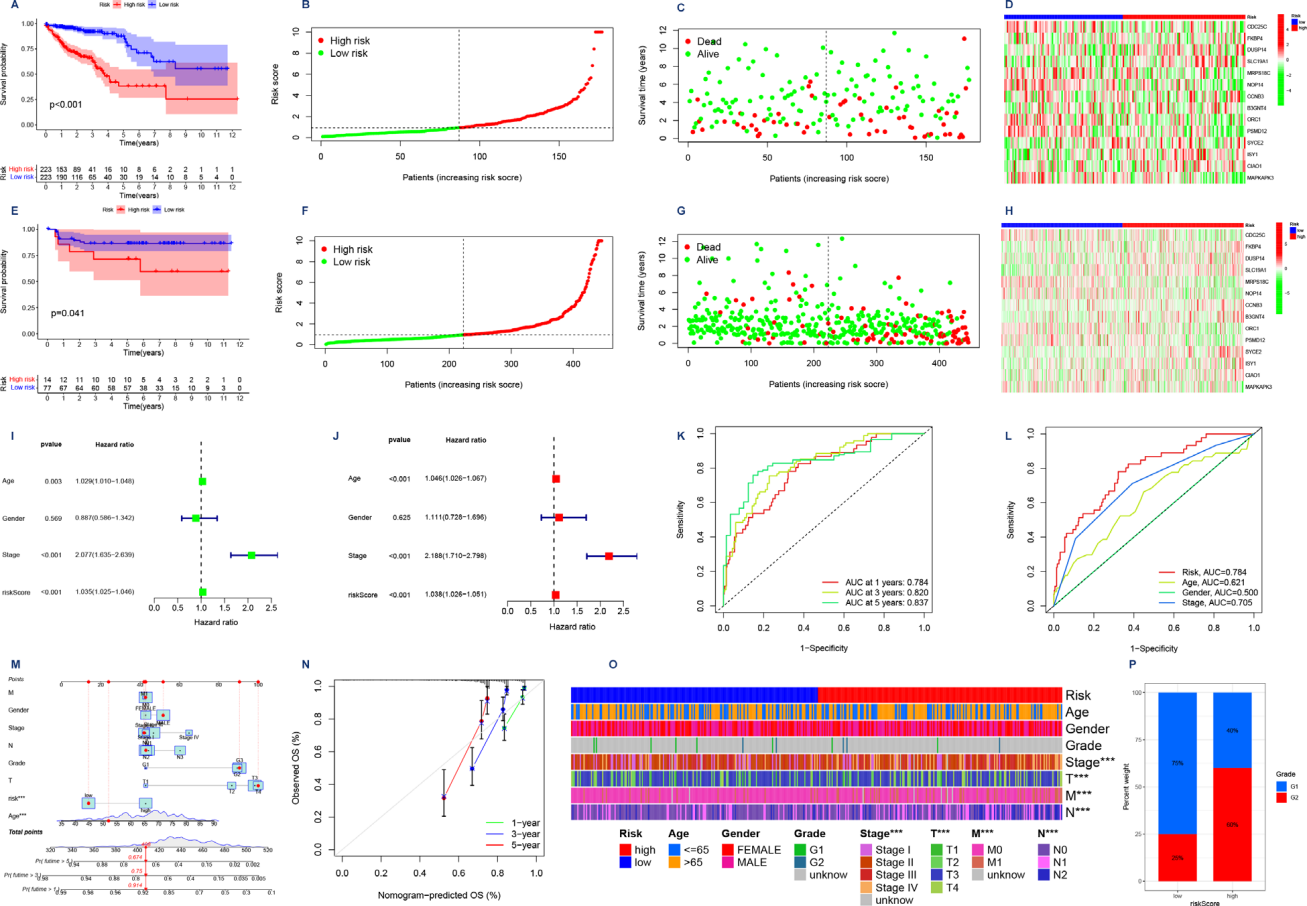
**(A,E)** Survival analyses were conducted on patients from the TCGA database and the GEO database, where they were categorized into high-risk and low-risk groups based on their risk scores. The Kaplan Meier survival curves depict the observation time on the horizontal axis and the corresponding survival rate on the vertical axis. **(B,F)** Risk curves. Low-risk patients are represented by green dots, while high-risk patients are indicated by red dots in terms of their risk scores. **(C,G)** Survival status graphs that have ranked patients in order of risk. The horizontal coordinate indicates the number of patients with increasing risk values from left to right; the vertical coordinate indicates the survival time of the patients. **(D, H)** Risk assessment is facilitated by a risk heat map, where the 14 genes are categorized as either high-risk or low-risk. The risk level increases progressively from left to right along the horizontal axis. **(I)** Forest plot was generated to analyze one-way Cox regression, incorporating clinical variables such as risk scores. **(J)** Forest plot illustrating the incorporation of clinical factors, including risk score, in multifactor Cox regression analysis. **(K)** ROC curves for assessing the precision of the model were generated for 1-, 3-, and 5-year OS durations. The x-axis represents false positive rate (1-specificity), while the y-axis represents true positive rate (sensitivity) **(L)** Joint ROC curves are utilized to assess the model’s prognostic accuracy, with the x-axis representing the complement of specificity and the y-axis indicating sensitivity. **(M)** Column line plot of the scoring of a patient including gender, age, stage, and risk score finally yielding the survival rate of that patient for different survival times. **(N)** Model calibration curve. The x-axis depicts predicted survival, while the y-axis represents observed survival, thereby enhancing the precision of real-time predictions. **(O)** Heatmap illustrating the contrasting clinical characteristics observed in the low-risk and high-risk groups, highlighting their distinctiveness. **(P)** A bar chart illustrating the distribution of grades among patients categorized into high and low risk groups. The x-axis represents the risk score, while the y-axis indicates the percentage of weight.

We utilized the TCGA database as our training dataset for constructing the prognostic model. Following that, patients from both the TCGA and GEO databases were categorized into two groups based on their risk scores relative to the median value. Individuals with a risk score surpassing the median were classified as belonging to the high-risk group, while those below it were assigned to the low-risk group. Survival analyses were then conducted, and **Figures 4A, E** illustrates the obtained results. The survival curves exhibited a gradual decline in patient survival rates over time for both datasets. Importantly, statistical analysis revealed significant disparities in patient survival between the high-risk and low-risk groups within both TCGA (*P*<0.001) and GEO (*P*<0.05) datasets. These findings provide compelling evidence supporting our model’s ability to effectively discriminate between patients at different levels of risk.

Next, we generated risk curves (**Figures 4B, F**). The risk score plot was ranked according to the risk level of the patients, and the median was used as the dividing line to classify the patients into high and low-risk groups. An increasing trend in mortality with increasing risk was evident (**Figures 4C, G**), aligning with our expectations. Furthermore, through the visualization of a risk heatmap (**Figures 4D, H**), we observed elevated expression levels of genes such as CDC25C, MRPS18C, NOP14, ORC1, PSMD12, and MAPKAPK3 in low-risk patients - indicating their association with lower risks. Conversely, high-risk patients exhibited heightened expression levels of genes including FKBP4, DUSP14, SLC19A1, CCNB3, B3GNT4, SYCE2, ISY1, CIAO1 along with other genes - signifying their involvement in higher risks.

After conducting autonomous prognostic analyses, our predictive model was found to be an independent prognostic factor irrespective of other clinical indicators. Both uniCox and multiCox regression analyses demonstrated a significant correlation with *P*<0.001 (**Tables 1**, **2**; **Figures 4I, J**). Furthermore, we performed ROC analysis to validate the accuracy of the model (**Figure 4K**), which yielded an AUC value of 0.784 at one year, 0.820 at three years, and 0.837 at five years (AUC>0.5 indicates good accuracy). The combined ROC curves also indicated that the accuracy of our model is higher than that predicted by other clinical indicators, such as age, gender, staging (**Figure 4L**).

**Table 1:** Results of the univariate cox regression analysis of the risk model.

ID	HR	HR.95L	HR.95H	*P* value
Age	1.02900163052518	1.01010337097291	1.04825346202302	0.00250369255642274
Gender	0.88667738970721	0.58600123968243	1.34162991505626	0.569231274302908
Stage	2.07733748988069	1.63511289033448	2.63916398211566	2.15039821891628e-09
riskScore	1.03542649394191	1.02489415710796	1.04606706645894	2.49320557708183e-11

**Table 2:** Results of the multivariate cox regression analysis of the risk model.

ID	HR	HR.95L	HR.95H	*P* value
Age	1.04623438153006	1.02596019419357	1.06690921079641	5.98434218125019e-06
Gender	1.11129733049112	0.72801009644269	1.69637998537552	0.624842205723537
Stage	2.18763208448807	1.71021464187516	2.79832368399929	4.6081790404592e-10
riskScore	1.03835669719359	1.0261119593351	1.05074755322551	5.00628730256291e-10

We proceeded to generate a column chart (**Figure 4M**) by assigning scores to various patient indicators, such as gender, age, stage, and risk score. These scores were then aggregated to obtain a composite score. By analyzing this chart, we could accurately determine the patient’s survival rate at different time intervals. For instance, if a patient had a composite score of 408, their survival rates would be 0.914 for over one year, 0.75 for over three years, and 0.674 for over five years. This column chart enabled us to make more precise predictions regarding the patient’s likelihood of survival. To further enhance prediction accuracy, we also made adjustments to the model depicted in **Figure 4N** where the yearly survival rate was set at 0.674.

### Importance of the risk score model in clinical practice

We performed an analysis of the clinical characteristics of both high-risk and low-risk groups, as depicted in **Figure 4O**. The findings reveal a significant discrepancy in T, M, N staging between these two groups (*P*<0.001). Additionally, **Figure 4P** showcases a noticeable differentiation in patient grading within the high-risk and low-risk classifications.

### Assessment of the correlation between risk scores and immune infiltration

**Figure 5:**
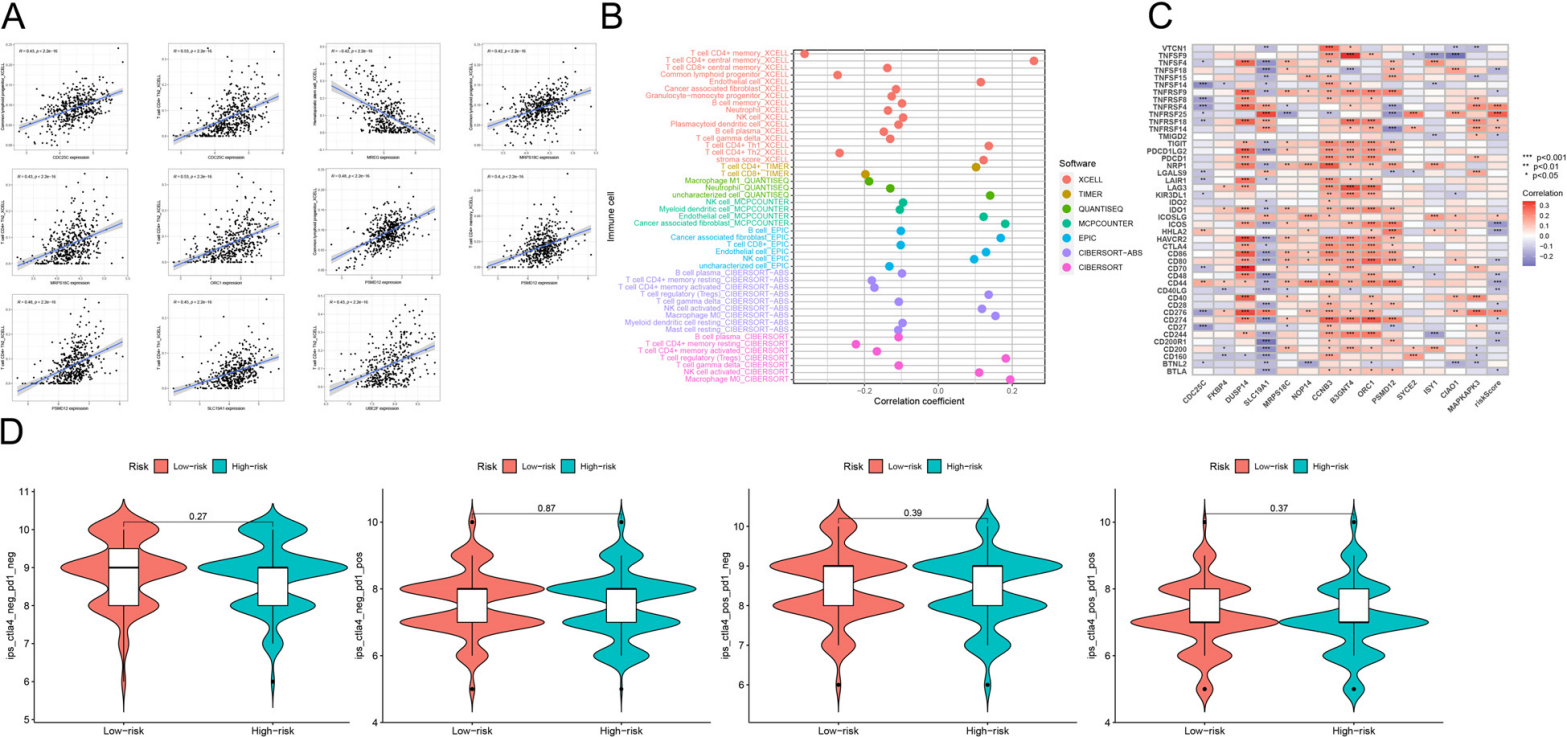
**(A)** Scatterplot analysis of correlation depicting the relationship between immune cells and genes utilized in model construction. **(B)** Correlation analysis of the correlation between genes used in constructing the model and immune cells using seven different quantitative methods for immune infiltration estimation, including TIMER, xCell, quanTIseq, MCP counter, EPIC, CIBERSORT-ABS and CIBERSORT. **(C)** The constructed risk score model is evaluated using GSVA analysis to determine the association between various immune checkpoints. **(D)** Graphical representations depicting the four different immunotherapy treatments administered to both high-risk and low-risk groups. These treatment protocols consist of combinations such as IPS-CTLA4-negative-PD1-negative, IPS-CTLA4-negative-PD1-positive, IPS-CTLA4-positive-PD1-negative, and IPS-CTLA4-positive-PD1-positive. ***p<0.001, **p<0.01, *p<0.05.

We used CIBERSORT and TIMER to investigate the association between genes involved in model construction and immune cells. As shown in **Figure 5A**, the expression levels of CDC25C and MRPS18C were positively correlated with common lymphoid progenitor cells and CD4+Th2 cells (*P*<0.05). Moreover, MREG expression exhibited a positive correlation with hematopoietic stem cells (*P*<0.05), ORC1 expression was positively correlated with CD4+Th2 cells (P<0.05), PSMD12 was also positively correlated with the expression of common lymphoid progenitor cells, CD4+T memory cells and CD4+Th2 cells (*P*<0.05). Additionally, SLC19A1 expression demonstrated a positive correlation with CD4+ Th1 cells (*P*<0.05), while UBE2F expression indicated a positive correlation with CD4+ Th2 cells (*P*<0.05). **Figure 5B** illustrates the relationship between different immune cell types and risk scores predicted by various software tools. Furthermore, **Figure 5C** showcases the connection between different immune checkpoints and our constructed risk score model. In our model genes, DUSP14,CCNB3, and B3GNT4 were positively correlated with most of the immune checkpoint genes (*P*<0.05,coef >0), while CDC25C was negatively correlated with most of the immune checkpoint genes (*P*<0.05,coef<0). Lastly, **Figure 5D** demonstrates that there were no statistically significant differences among the four immunotherapy regimens (IPS-CTLA4-negative-PD1-negative, IPS-CTLA4-negative-PD1-positive, IPS-CTLA4-positive-PD1-negative, and IPS-CTLA4-positive-PD1-positive) within both high-risk and low-risk groups (P>0.05), indicating no variation in the effectiveness of immunotherapy across these regimens.

### Correlation of risk score models with somatic variance

**Figure 6:**
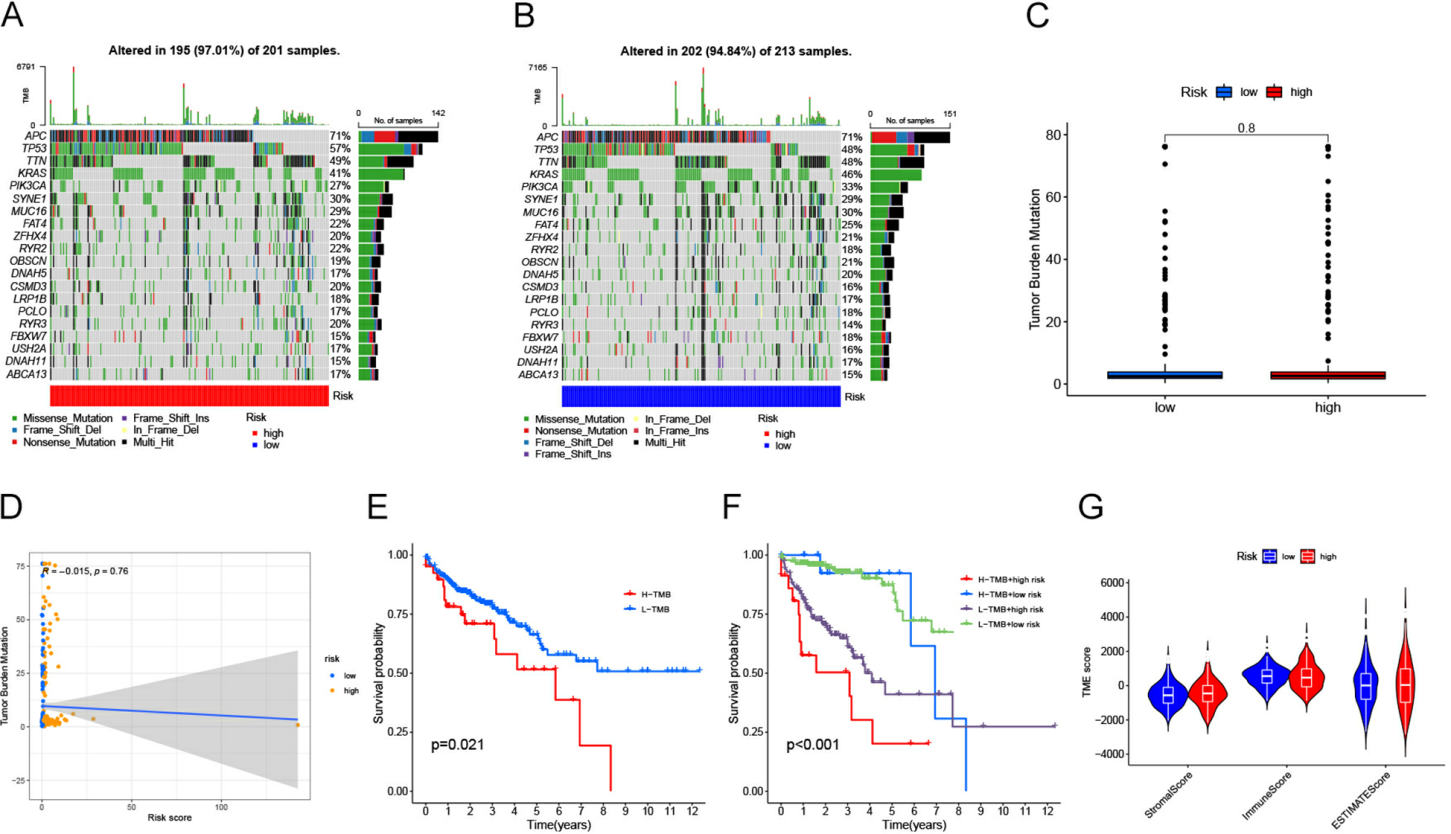
**(A)** Waterfall diagram illustrating the mutations observed in each patient sample from the high-risk group of individuals with bowel cancer. The plot includes information on mutation characteristics and tumor mutational burden (TMB). **(B)** Waterfall diagram illustrating the frequency of gene mutations observed in bowel cancer patient samples belonging to the low-risk group. The plot also includes information on specific mutation characteristics and TMB. **(C)** A box line plot is presented to illustrate the variation in tumor mutational load (TMB) between the high and low risk groups. The horizontal axis represents risk levels, while the vertical axis displays tumor mutational load. **(D)** Scatterplot illustrating the correlation between risk score and TMB. The x-axis represents risk score, while the y-axis represents tumor mutational load. **(E)** A Kaplan-Meier diagram illustrates the contrast in survival outcomes between groups categorized by their tumor mutational load, with time on the horizontal axis and probability of survival on the vertical axis. **(F)** Survival odds were plotted against survival times using Kaplan-Meier curves obtained from stratified survival analysis. **(G)** Graphical representation of violin plot. The x-axis represents matrix/immunity/estimated scores, while the y-axis represents TME scores.

We further examined the genetic mutations in patients with colorectal cancer who are at a higher risk compared to those at a lower risk. Waterfall diagrams, as shown in **Figures 6A, B**, provide detailed information on the mutations observed. The analysis presented in **Figure 6C** did not reveal any significant differences in tumor mutation burden (TMB) between the two groups (P>0.05). Similarly, **Figure 6D** suggests that there is no notable correlation between risk score and TMB (P>0.05). However, according to **Figure 6E**, patients with high tumor mutation loads have a significantly lower survival rate compared to those with low tumor mutation loads (*P*<0.05). It is worth noting that individuals with lower tumor mutation loads tend to have better survival outcomes. In addition, the results of further stratified survival analysis (**Figure 6F**) showed that patients with low TMB in the high-risk group showed a survival advantage, and the difference in survival curves between the two groups of high and low TMB in the low-risk group was not very significant. However, it is obvious that the overall survival of patients in the low-risk group is significantly longer than that in the high-risk group, both in the high TMB group and the low TMB group. Finally, the stromal/immune/estimated scores were calculated using the ESTIMATE algorithm as depicted in **Figure 6G**.

### Correlation of risk scoring models with biological function

**Figure 7:**
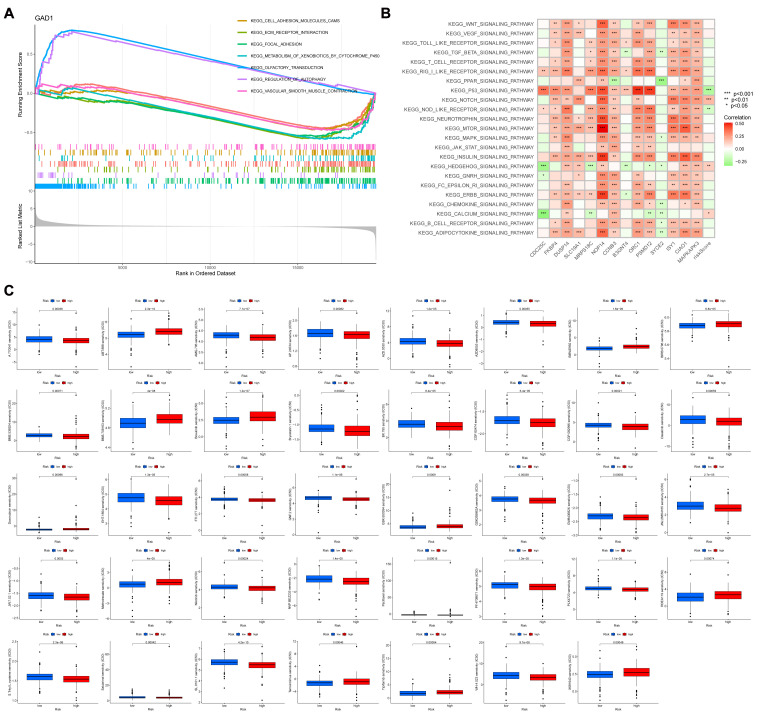
**(A)** GSEA shows the enrichment of seven pathways. Horizontal coordinates are the sorted values of the dataset, and vertical coordinates are the enrichment scores and the amount of genes sorted. **(B)** Revealing the association between diverse signaling pathways and the risk score model is demonstrated through GSVA analysis. **(C)** A box line plot is utilized to illustrate the variation in sensitivity levels among 39 drugs, specifically comparing high and low risk groups. The horizontal axis represents the level of risk, while the vertical axis indicates the degree of sensitivity. ***p<0.001, **p<0.01, *p<0.05.

GSEA was used to identify potential regulatory mechanisms (**Figure 7A**). The results of enrichment analysis showed a significant enrichment of olfactory signaling pathway, autophagy regulation pathway in the high expression group of GAD1 gene, and cell adhesion pathway, extracellular matrix receptor interactions pathway, adhesion plaque pathway, drug metabolism-cytochrome P450, and vascular smooth muscle contraction pathway in the low expression group. **Figure 7B** shows the correlation between different signaling pathways and the risk scoring model: the P53 signaling pathway, oligomerized nucleotide-binding structural domain-like receptor signaling pathway, NOTCH signaling pathway, HEDGEHOG signaling pathway, and calcium ion signaling pathway correlated with the risk scoring model (*P*<0.05). And we explore the sensitivity of risk-scoring models to drugs and immunotherapy regimens by using the pRRophetic algorithm (**Figure 7C**). We observed significant differences (*P*<0.05) in the sensitivities of all 39 drugs tested between the high- and low-risk groups, with a majority of the drugs exhibiting lower sensitivities in the high-risk group compared to the low-risk group (the low-risk group displayed a lower IC50).

## Discussion

The progression of CRC is characterized by slow growth and high heterogeneity, which presents challenges in determining the most appropriate immunotherapy for individual patients. Previous studies have demonstrated a correlation between immune cell infiltration in CRC and patient prognosis (16). WGCNA is an efficient method that accurately analyzes multiple genes, enabling the construction of networks to explore the relationship between different genes and clinical manifestations (17). Although several studies have utilized WGCNA to develop prognostic prediction models, there remains a lack of comprehensive models capable of assessing both patient prognosis and response to immunotherapy. To address this gap and identify personalized therapies for CRC patients, we conducted immune infiltration analysis on 514 samples and constructed a prognostic model using WGCNA combined with LASSO-Cox regression.

In this study, we successfully demonstrated the efficacy of our prognostic prediction model. By employing the WGCNA method and integrating data from the TCGA and GEO databases, our model consistently obtained AUC values greater than 0.70 in receiver operating characteristic (ROC) curves for the prediction of overall survival (OS) time at 1, 3, and 5 years. In addition, in the combined ROC analysis, the area under the curve (AUC) of different predictors showed that the risk score of our model had significantly better predictive performance than Age,Gender and stage (Risk: AUC=0.784,Age: AUC=0.621,Gender: AUC=0.500,Stage: AUC=0.705). Additionally, we have noticed strong associations between the risk-assessment model and different variables, such as the tumor microenvironment, biological function, as well as response to chemotherapy and immunotherapy. These findings further validate the predictive capabilities of our model and may serve as a valuable reference for anticipating immunotherapy outcomes in patients.

The tumor microenvironment consists of both tumor cells and immune cells that penetrate into the tumor. The interaction between these two cell types significantly influences the development of tumors (18). Our study analyzed the extent of infiltration by different immune cells in each sample, and through analysis of variance, we concluded that compared to normal samples, tumor samples exhibited higher rates of infiltration by activated mast cells, neutrophils, etc. Conversely, there was a lower rate of infiltration by resting dendritic cells, resting mast cells, M2 macrophages, and eosinophilic granulocytes. Tumor-associated macrophages and neutrophils are generally believed to promote tumor growth; however, their plasticity and polarization within different tumor microenvironments can lead to the formation of distinct phenotypes with varying roles in tumors (19). Previous studies have shown that CD4+ T-cells secrete cytokines to support anti-tumor immunity generated by CD8+ T-cells. Additionally, eosinophils may be associated with controlling CD4+ T-cell activity while exhibiting an inverse correlation with regulatory T-cell characteristics (20). Examining these populations of immune cells offers a more comprehensive comprehension of the correlation between the tumor microenvironment and the progression of tumors.

We performed clustering analysis on immune cell-related genes to construct a co-expression network, through which we identified fourteen genes that were utilized in our model development. Among these genes, CDC25C, MRPS18C, NOP14, ORC1, PSMD12, and MAPKAPK3 were classified as low-risk genes. Conversely, FKBP4, DUSP14, SLC19A1, CCNB3,B3GNT4,SCEY2,ISY1,and CIAO1 were categorized as high-risk genes. In an independent investigation that centers on the development of a prognostic forecasting model for individuals with colon cancer, DNA repair-associated genes (DRGs) were examined, CDC25C, which is involved in cell cycle activity, was found to be a low-risk gene involved in the regulation of the G2/M transition cell cycle checkpoint and DNA damage repair (21). The CDC25C gene was identified as one of the key genes in both the prediction of colon adenocarcinoma by anchorage-dependent cell death-related genes as well as in prognostic models of colon cancer (22–24). This corroborates the application of CDC25C gene in prognosis. Similarly, PMSD12 was also considered as a low-risk gene, which can be used as a feature to predict the prognosis of colon adenocarcinoma in amino acid metabolism-related models (25). Zhang et al. found that there was a large amount of NOP14 mRNA in the tissues of colon cancer patients, which was considered to be a protective prognostic factor for colon cancer patients (26). Zhu et al. mentioned that NOP14 is important in cell proliferation, cell metastasis, cell apoptosis and other tumor progression is important (27). ORC1 plays an important role in epigenetic regulation by modifying histones. Chromatin remodeling or acetylation in CDC6/ORC1 may delay DNA replication and promote the development of colorectal cancer (28). All of the above studies confirmed the prognostic value of low-risk genes in colon cancer. Regarding high-risk genes, DUSP14 has been identified as a target gene of ATF3, which has an impact on the development of CRC; therefore it is closely associated with poor prognosis in CRC patients (29). Meanwhile, FKBP4 has been found to have multiple functions in various human diseases associated with hormone-dependent, stress-related and neurodegenerative changes (30–32). And FKBP4 expression is upregulated in most cancer types such as breast cancer, squamous lung cancer, etc., which means the high expression of FKBP4 may be closely related to tumorigenesis (33). In addition, as part of the spliceosome C complex, higher expression level of ISY1 was associated with better prognosis in cervical cancer, which is contrary to our findings and may be related to the biological characteristics of cancer, tumor microenvironment, cancer biology, and differences in gene expression profiles, etc., which need to be further investigated (34). Abnormal glycosylation is a prevalent form of post-translational modification that contributes to the diversity observed in CRC. Based on its involvement in constructing glycemic risk prediction models, B3GNT4 has been recognized as a high-risk gene linked to unfavorable prognosis (35). All of these findings provide substantial theoretical support for our developed prognostic model.

All of these findings provide substantial theoretical support for our developed prognostic model.

Immunological examinations were conducted to investigate patient responsiveness toward chemotherapy and immunotherapy. The inhibitory surface molecules that are highly expressed on the surface of exhausted T cells to prevent their activation are defined as immune checkpoints (36), which are key molecules in the regulation of immune response and are involved in the maintenance of immune tolerance and the regulation of immune response (37). Normally, immune checkpoints inhibit the activity of T cells through negative regulation to prevent T cells from attacking normal tissues (38). However, many tumor cells are taking advantage of this property to inhibit T cells by upregulating immune checkpoints to achieve immune escape, enabling them to survive and proliferate *in vivo* (39). Recently, immune checkpoint regulation through targeted immunotherapy, known as ICB, has demonstrated promising effectiveness in cancer treatment (40). Our study identified prognostic models that correlated with various immune checkpoints such as TNFSF18, TNFRSF4, TNFRSF9, TNFRSF14, TNFRSF18, TNFRSF25, TMIGD2, CD28, CD44, CD48, CD80, CD244, CD274, CD276, CD200R1, CD40LG, ICOSLG, ICOS, HHLA2 and BTLA. We found that TNFRSF4, ICOSLG and TNFRSF25 were positively correlated with the risk score (*P*<0.05,coef>0), while ICOS and HHLA2 were negatively correlated with the risk score (*P*<0.001,coef<0). Li et al. also reported similar findings while constructing a prognostic model for CRC (41). It has been proposed that the upregulation of ICOS/ICOSLG expression could potentially play a role in the advancement of atypical cytology, transitioning from low-grade colorectal lesions to high-grade lesions and ultimately leading to CRC. This finding aligns with our analysis results (42). To evaluate the response to ICB treatment we employed TMB and TIDE scoring methods. Generally speaking, patients with high TMB respond better to ICB therapy (43), whereas higher TIDE scores are associated with poorer response to ICB therapy (44). Therefore, TMB can affect patient survival by affecting the response to immunotherapy. However, in our study, the overall prognosis of the high TMB group was worse than that of the low TMB group (**Figure 6E**), and similar results were also found in the study of Chen et al. (45). According to the results of our further subgroup analysis, this may be because the main factor affecting the prognosis of patients is still the risk score (**Figure 6F**), which also showed similar results in the study of Jin et al. (46). In addition, our results showed that there was no significant difference in tumor mutation burden (TMB) and Tumor immunotherapy response score between the high and low risk groups (*P*>0.05), which may be due to the prognostic model constructed based on TCGA database samples. In real clinical conditions, most of these samples are treated with first - and second-line chemotherapy regimens, in which chemotherapy-sensitive patients are more likely to show better prognosis, which is independent of immunotherapy. Thus, although our prognostic model was based on immune-related genes, it was not directly related to TMB and Tumor immunotherapy response score. Additionally, four different immunotherapy regimens (IPS-CTLA4-neg-PD1-neg; IPS-CTLA4-neg-PD1-pos; IPS-CTLA4-pos-PD1-neg; IPS-CTLA4-pos-PD1-pos) showed no notable differences in treatment effects (P>0.05) during subsequent analyses.

The selected 39 drugs exhibited varying sensitivities between the high and low-risk groups (*P*<0.05). Notably, certain drugs such as S.Trityl.L.cysteine, SL.0101.1, WH.4023, JW.7.52.1, NVP.BEZ235, PF.4708671, JNJ.26854165, EHT.1864, GSK269962A, Dasatinib, BMS536924, Bryostatin.1, Ponatinib, AMG706, AZD0530, AZD8055 and GSK650394 revealed a higher level of sensitivity within the high-risk group while Temsirolimus, T ipifamib, X681640, RDEA119, Methotrexate, BMS708163, Bosutinib, BIRB0796, BIBW2992, S alubrinal, Nilotinib, Paclitaxel, PLX4720, FTI277, GNF2, GW843682X AP24534, AZD8055 and ABT888 showed higher sensitivities in the low-risk group. This disparity may potentially arise from variances in the physiological mechanisms exhibited by these distinct cohorts. Tyrosine kinase inhibitors (TKIs) were observed to be highly enriched among the drugs that demonstrated sensitivity in the high-risk group which may suggest a correlation with protein tyrosine kinase-associated pathways like IL6/JAK/STAT and PI3K/AKT where high-risk patients are at significant risk of developing diseases associated with these pathways.

Nevertheless, it is crucial to recognize specific constraints in our research that require consideration. Further validation of our model should be pursued by analyzing a substantial number of samples to effectively demonstrate its prognostic value. Moreover, conducting biological experiments is imperative for investigating the potential mechanisms underlying immune-related prognostic genes in CRC.

## Conclusion

In brief, our study has devised a risk assessment model that effectively forecasts the OS of colon cancer patients by incorporating immune-associated genes. This novel approach exhibits significant promise as a dependable technique for prognostic evaluation in the field of clinical practice. Moreover, we have identified significant distinctions between the low-risk and high-risk groups in terms of their clinical characteristics, immune checkpoint expression, infiltration patterns of immune cells, and drug sensitivity profiles. These groundbreaking findings provide valuable insights into exploring novel targets for immunotherapy in patients with colon cancer.

## Data Availability

The original contributions presented in the study are included in the article/supplementary material. Further inquiries can be directed to the corresponding author.
